# When Indian crabs were not yet Asian - biogeographic evidence for Eocene proximity of India and Southeast Asia

**DOI:** 10.1186/1471-2148-10-287

**Published:** 2010-09-17

**Authors:** Sebastian Klaus, Christoph D Schubart, Bruno Streit, Markus Pfenninger

**Affiliations:** 1 Department of Ecology and Evolution, Goethe University, Frankfurt am Main, Germany; 2 Biologie 1, University of Regensburg, Regensburg, Germany; 3 Biodiversität und Klima Forschungszentrum, Frankfurt am Main, Germany

## Abstract

**Background:**

The faunal and floral relationship of northward-drifting India with its neighboring continents is of general biogeographic interest as an important driver of regional biodiversity. However, direct biogeographic connectivity of India and Southeast Asia during the Cenozoic remains largely unexplored. We investigate timing, direction and mechanisms of faunal exchange between India and Southeast Asia, based on a molecular phylogeny, molecular clock-derived time estimates and biogeographic reconstructions of the Asian freshwater crab family Gecarcinucidae.

**Results:**

Although the Gecarcinucidae are not an element of an ancient Gondwana fauna, their subfamily Gecarcinucinae, and probably also the Liotelphusinae, evolved on the Indian Subcontinent and subsequently dispersed to Southeast Asia. Estimated by a model testing approach, this dispersal event took place during the Middle Eocene, and thus before the final collision of India and the Tibet-part of Eurasia.

**Conclusions:**

We postulate that the India and Southeast Asia were close enough for exchange of freshwater organisms during the Middle Eocene, before the final Indian-Eurasian collision. Our data support geological models that assume the Indian plate having tracked along Southeast Asia during its move northwards.

## Background

The Indian Subcontinent has a biogeographic key position between Africa, Madagascar and Eurasia. It has been involved in faunal exchange between the continents during the Late Cretaceous and Paleogene by either serving as a biotic ferry for a Gondwana fauna [1-4], or as a target [5-7], stepping-stone [8,9] or source [8,10] for dispersal. For all biogeographic scenarios involving the Indian Subcontinent, the assumed geological setting is of major importance; especially the time of fragmentation of Eastern Gondwana, the position of Greater India on its move northwards relative to the adjacent continents, and the timing of the Indian-Eurasian collision.

**Figure 1:**
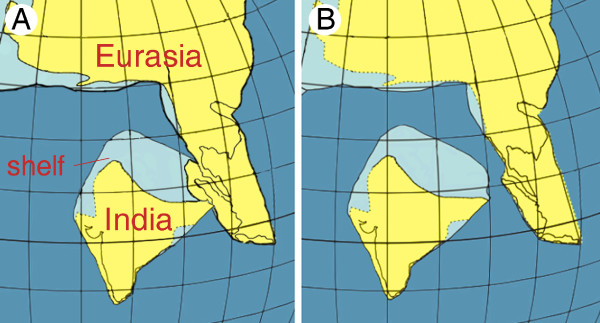
**Eocene position of India**. Relative position of the Indian and Southeast Asian continental plates during the Middle Eocene (45 Ma) according to Ali and Aitchison [17], with the position of Southeast Asia based on Hall [68], and the distribution of land and sea after Smith et al. [69]. A: based on the motion path of the Indian Subcontinent of Acton [19]. B: based on the motion path of Schettino and Scotese [18].

The standard geological view dates the Indian-Eurasian collision to the Paleocene/Early Eocene at 50-55 Ma [11-13]. However, there is stringent evidence from both geological data and plate modeling approaches for a much later collision around 35 Ma, preceded by collision with an intraoceanic island arc around 55 Ma [14-17]. Ali and Aitchison [17] presented two hypotheses on the location of India during its Eocene northward movement. The first is based on the motion path of the Indian Subcontinent according to Schettino and Scotese [18] and assumes India in an isolated position until final contact with Tibet (Fig. 1B). The second hypothesis, using the motion path of Acton [19], reconstructs India in close proximity to Southeast Asia from Eocene to Oligocene, possibly tracking along Sumatra, the Malay Peninsula and Burma, and allowing terrestrial connections (Fig. 1A). Depending on which of the two geological models fits better to the actual Indian motion path, one should expect different biogeographic and phylogenetic patterns as both models allow different spatial and temporal dispersal pathways. In particular, the geological history should be reflected by taxa with limited marine dispersal capabilities, e.g., terrestrial non-volant or limnic organisms.

Freshwater crabs are considered to be good indicators of zoogeographic patterns [20]. Their ontogeny is completely independent from the marine realm, and they are thought to be drainage basin specific [21,22]. Although freshwater crabs can to some extent be saltwater tolerant under laboratory conditions [23] and may incidently have surmounted shorter marine passages by rafting [24], there is no evidence for transoceanic long-distance dispersal of freshwater crabs [25,26].

The palaeotropic freshwater crab family Gecarcinucidae occurs from the Indian Subcontinent to Australia, including tropical East Asia, the Indonesian and Philippine archipelagos, the Moluccas and New Guinea [9,24]. This distribution pattern makes the Gecarcinucidae a well suited model taxon to estimate timing, direction, and possible mechanisms of faunal exchange between Southeast Asia and India during the Cenozoic.

We construct a robust molecular phylogeny of the Gecarcinucidae, provide molecular clock estimates and use these data to reconstruct ancestral ranges. Specifically, we test the fit of the respective biological data to the recently developed geological models of the Indian-Eurasian collision.

## Results

### 0.1 Phylogenetic analyses and molecular clock estimates

**Figure 2:**
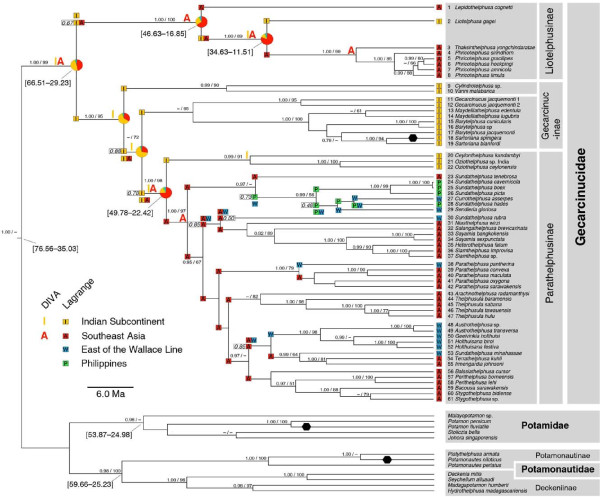
**Phylogeny of the Gecarcinucidae**. Maximum clade credibility tree of the Gecarcinucidae based on Bayesian inference of 16 S rDNA and histone H3 sequences. At nodes both the posterior probabilities (> 0.90) of the Bayesian analysis and the bootstrap values (> 50) of the maximum likelihood analysis are given. Hexagons indicate the three fossil calibration points (see text), time estimates in square brackets represent the 95% confidence interval. Range inheritance as reconstructed by Lagrange is visualized by letters in colored squares, in the case of alternative reconstructions within two log-likelihood units the relative probability of the better reconstruction is added in a square box. Ancestral area reconstruction by BayesTraits is represented by pie charts with the ranges color-coded as for the Lagrange analysis.

The inferred phylogenetic topology of the Gecarcinucidae is congruent in all deeper splits to a previously published phylogeny [24]. Also our Maximum Likelihood and Bayesian analyses do not conflict at the basal splits within the Gecarcinucidae. Within the subfamily Parathelphusinae our analyses retrieve the lineages identified by Klaus et al. [24], although the relationship between these lineages have low support and differ between both of our phylogenetic approaches. The Maximum Likelihood analysis supports the sister group relationship of the genus *Parathelphusa *with the Malesian-Australian group (in line with Klaus et al. [24]). Monophyletic and well supported are two subfamilies, the Liotelphusinae and the Parathelphusinae, while the third subfamily Gecarcinucinae is paraphyletic (Fig. 2).

The time estimate for the root of the Gecarcinucidae ranges from the Oligocene to the Cretaceous with a 95% highest posterior density credibility interval (HPD) of 66.51-29.23 Ma. For biogeographic inference of the early evolution of the Gecarcinucidae, two nodes are of major importance: the most recent common ancestor (MRCA) of the Liotelphusinae (95% HPD 46.63-16.85 Ma) and the MRCA of the Parathelphusinae (95% HPD 49.78-22.42 Ma), as at these nodes Indian and Southeast Asian species split. The divergence time estimation for both nodes shows a large 95% credibility interval. Therefore, we tested different temporal models that assume divergence before, during or after the final Indian-Eurasian collision around 35 Ma. A Middle Eocene origin for the MRCA of both Parathelphusinae and Liotelphusinae is strongly supported, thus most likely predating the final Indian-Eurasian collision (Table 1).

**Table 1:** Testing of temporal models

	ln [post. prob.] ± SE	Para 45 Liot 45	Para 35 Liot 35	Para 25 Liot 25	Para 45 Liot 35	Para 35 Liot 45	Para 35 Liot 25	Para 25 Liot 25
Para 45 Liot 45	-14285.01 ± 0.19	-	0.95*	1.73***	0.66*	1.24**	2.74***	1.34**
Para 35 Liot 35	-14287.19 ± 0.15	-0,95	-	0.78*	0.78*	0.29	1.80***	0.39
Para 25 Liot 25	-14288.99 ± 0.17	-1.73	-0.78	-	-1.06	-0.49	1.02**	-0.39
Para 45 Liot 35	-14286.54 ± 0.16	-0.66	-0.78	1.06**	-	0.58*	2.08***	0.67*
Para 35 Liot 45	-14287.86 ± 0.17	-1.24	-0.29	0.49*	-0.58	-	1.51***	0.10
Para 35 Liot 25	-14291.33 ± 0.15	-2.74	-1.80	-1.02	-2.08	-1.51	-	-1.41
Para 25 Liot 35	-14288.09 ± 0.17	-1.34	-0.39	0.39	-0.67	-0.10	1.41**	-

Testing of the temporal models with different time constraints in million years for the most recent common ancestor (MRCA) for the subfamilies Parathelphusinae (Para) and Liotelphusinae (Liot). The first three models (first row) constrain the age of both MRCAs before, during and after the assumed final collision of India and Eurasia; the next four models assume different ages for the MRCA of Parathelphusinae and Liotelphusinae, i.e., either before or during collision of plates, or during and after this event. The natural logarithm of the models posterior probabilities and its standard error, and the common logarithm of the Bayes factor between models are given. Models in the left column are tested against the models in the first row (* substantial, ** strong, *** very strong support for one model over another). The first model, constraining the MRCA of both subfamilies into the Middle Eocene (45 Ma), i.e., before the final Indian-Eurasian collision, is superior to all other models.

### 0.2 Origin of the Gecarcinucidae and their subfamilies

The reconstruction of the ancestral range of the Gecarcinucidae according to the Bayesian inference of ancestral character states (BayesTraits) remains equivocal. However, none of the present ancestral area reconstructions of the BayesTraits analysis has support over alternative reconstructions (log_10 _Bayes factor < 0.48). This points out the necessity to test the significance of the favoured reconstruction when using this method for biogeographic purposes. Also dispersal-vicariance analysis (DIVA) does not assign the root of the Gecarcinucidae unequivocally to one area, but assumes a widespread range comprising both India and Southeast Asia.

Therefore, we also applied a likelihood approach to geographic range evolution based on dispersal, extinction and cladogenesis (DEC-model) to investigate if dispersal from India or from Southeast Asia can better explain the present range patterns. Of the three different dispersal-models calculated (Table 2), the Akaike information criterion (AIC) and Akaike weight support the H_*I→A*_-model (allowing only dispersal from India to Asia) with a dispersal rate of 3.90 × 10^-3 ^and an extinction rate of 3.19 × 10^-4^. Thus, dispersal from India to Southeast Asia gives the best explanation of the present data. The resulting reconstruction is shown in Fig. 2.

**Table 2:** Testing of dispersal models

Model	log likelihood	AIC	*w*
H_*I→A*_	-48.88	111.76	0.975
H_*A→I*_	-50.51	115.20	0.019
H_0_	-50.09	116.18	0.006

Different dispersal models for the Gecarcinucidae that were tested in Lagrange to investigate the direction of faunal exchange between India and Southeast Asia. Given are the global maximum likelihood at the root node, the AIC, and the Akaike weight (*w*) of the respective dispersal models. The H_0 _model is without any dispersal constraint, H_*I→A*_only allows dispersal from India to Southeast Asia, H_*A→I *_only from Southeast Asia to India.

The Indian Subcontinent consistently emerges in all three biogeographic methods as the most likely ancestral area of the MRCA of the subfamilies Gecarcinucinae and Parathelphusinae. The Indian range is retained throughout the paraphyletic Gecarcinucinae and passed over to the Parathelphusinae, with the latter reaching Southeast Asia only once. Within this parathelphusine Southeast Asian clade a complex pattern of range evolution is reconstructed, with several transitions of the Wallace Line.

For the MRCA of the Liotelphusinae the ancestral range with the best likelihood value (according to the DEC-model) comprises both Indian and Southeast Asian areas (relative probability: 0.67). The Indian area is lost in the ancestor of *Lepidothelphusa cognetti*, whereas the Indian-Southeast Asian area was retained in the Liotelphusinae until the divergence of the genus *Liotelphusa *(95% HPD 10.79-34.30 Ma). In contrast, DIVA reconstructs an Asian MRCA and secondary dispersal to India followed by vicariance.

## Discussion

In agreement with previous results [9,27], the present data reject an early Gondwana distribution for the Gecarcinucidae, both concerning tree topology and time estimates. The Gondwana hypothesis for the Old World freshwater crabs does not hold against the freshwater crab fossil record (being not older than Oligocene/Miocene [28,29]), and is not in accordance with divergence time estimates [27] and phylogenetic reconstructions that do not reflect the break up of Eastern Gondwana [9,24,27]. Moreover, the initial separation of western (South America and Africa) and eastern Gondwana predates the first fossils of heterotreme marine brachyurans by approximately 50 million years.

The hypothesis of an African origin of the Gecarcinucidae can not be validated with the present data. The presumed dispersal to the Indian Subcontinent via Madagascar and the Seychelles Bank during Oligocene low sea level [9] can be rejected as the Gecarcinucidae do not nest within a paraphyletic African Potamonautidae; in contrast, the freshwater crabs of Madagascar and the Seychelles are monophyletic with the Potamonautidae. Also the estimated divergence times of the most basal splits within the Gecarcinucidae are likely to predate the Oligocene. Nevertheless, the age of separation between East African *Deckenia mitis *and *Seychellum alluaudi *from the Seychelles (95% HPD 26.88-8.08 Ma) and the age of the deepest split within the potamonautid subfamily Deckeniinae (95% HPD 48.54-19.29 Ma) do not conflict with the hypothesis of these Oligocene stepping stones. These could have enabled independent colonization of Madagascar and the Seychelles from Africa, as it was already proposed for amphibians and cichlid fishes [30,31], and is additionally supported by the reconstruction of Paleogene ocean currents [32]. The age of the root of the present phylogeny, and the estimated age of the Gecarcinucidae fit very well into the period of intensive brachyuran radiation, i.e., Upper Cretaceous to Early Eocene, that is apparent from the fossil record [33]. We hypothesize that during this time span the marine ancestor of the Old World freshwater crabs might have invaded the limnic habitat from the ancient Tethys Ocean. According to our biogeographic reconstruction, at least for the clade [Gecarcinucinae + Parathelphusinae], this transition occurred most likely at the shores of the Indian Subcontinent.

The results of the temporal model testing indicate that the separation of Indian and Southeast Asian species within the subfamily Parathelphusinae more likely occurred in the Middle Eocene than in the Oligocene, and according to the biogeographic reconstructions, the Indian subcontinent was by far more likely the area of origin for both Gecarcinucinae and Parathelphusinae than Southeast Asia. Provided that the final collision of India and the Tibet-part of Eurasia took place in the Late Eocene [16], we must either assume that gecarcinucid freshwater crabs surmounted a marine barrier to reach Southeast Asia, or that a land connection between India and Southeast Asia existed. As transoceanic long distance dispersal of freshwater crabs appears highly unlikely [25,26], we conclude that India and Southeast Asia were at least very close. An early to Middle Eocene exchange of freshwater organisms is best explained by the Indian motion path of Acton [17,19] which implies direct contact of plates and probably would allow terrestrial connections (Fig. 1A). In contrast, the position of the Indian Subcontinent based on Schettino and Scotese [17,18] hypothesizes an open marine strait of about 500 km between the Indian plate and Eurasia (Fig. 1B). In fact, the exact position of western Southeast Asia during the Eocene, decisive for the present considerations, is not definitively solved and could be placed slightly eastward (J.R. Ali, pers. comm.). As in the Parathelphusinae, a Middle Eocene age for the MRCA of the Liotelphusinae is the preferred model, also predating the Indian-Eurasian collision. According to the DEC-model of range evolution the MRCA inhabited both Indian and Southeast Asian areas, while DIVA and the BayesTraits analysis favours a Southeast Asian origin (with the reservation that the latter is not superior to alternative reconstructions, see above). Several scenarios could explain this pattern: The liotelphusine ancestor could have had a widespread range comprising India and Southeast Asia during the Middle Eocene, as reconstructed in Lagrange. This would fit to the geological model that assumes contiguity of the Indian plate and Southeast Asia during that time. Alternatively, early diverging Indian representatives are not sampled in the present data set or could have gone extinct, raising the possibility that the ancestor of the genus *Liotelphusa *dispersed back to the Indian Subcontinent after the final Indian-Asian collision. Also, an independent colonization of the freshwater habitat can not be excluded with certainty. To solve the history of this subfamily with confidence, a larger sampling of the enigmatic Indian liotelphusine species will be necessary. Eocene faunal exchange between India and Southeast Asia could not only be facilitated by the paleogeographic proximity of the Indian Subcontinent and Southeast Asia, but also favored by equal climatic conditions. As indicated by coal deposits, both areas most probably have been dominated by closed-canopy megathermal rain forests during the Early Eocene, and displayed an everwet tropical climate [8,34]. This Indian-Southeast Asian moist corridor was already assumed to enable dispersal of Indian floral elements into Southeast Asia, corroborated by palynological evidence [8,34,35].

Several Asian faunal or floral elements are claimed to be Gondwana relics, entering Asia via the northwards drifting Indian Subcontinent, i.e., the ''out-of-India' scenario [2,4,36]. These comprise ranoid and hyloid frogs [37,38], gymnophionans [39,40], agamid lizards [41], cichlids [42], aplocheiloid fishes [43], the Asian arowana [44], Crypteroniaceae [45] and Dipterocarpaceae [46,47]. In contrast, we present here for the first time evidence that the Gecarcinucidae, a taxon that is definitely not a Gondwana element, originated on the Indian Subcontinent, at least with the subfamily Gecarcinucidae, and dispersed into Southeast Asia during the Middle Eocene.

The early range evolution of gecarcinucid freshwater crabs exemplifies that the recently developed geological models of the Indian-Eurasian convergence are generally in accordance with biogeography [17]. Very likely this direct Eocene biotic exchange between India and Southeast Asia, possibly in both directions, accounts for the distribution patterns of a large set of organisms and probably contributed profoundly to the vast biodiversity of present South and Southeast Asia.

## Conclusions

The Asian freshwater crab family Gecarcinucidae most probably originated on the Indian Subcontinent and dispersed secondarily into Southeast Asia. Within the subfamilies Gecarcinucinae and Liotelphusinae Indian and Southeast Asian species split in the Middle Eocene, before the final Indian-Eurasian collision. We postulate that the Indian plate and Southeast Asia were close enough for exchange of freshwater organisms during that period. Our data support geological models that assume the Indian plate having tracked along Southeast Asia during its move northwards.

## Methods

### 0.3 Phylogenetic analysis and molecular dating

Gene sequences of the nuclear encoded histone H3 gene and the partial mitochondrial 16 S rDNA were retrieved from Genbank and originated from previous studies on the phylogeny of the Gecarcinucidae (AM234635, AM234637, AM234640, AM234641, AM234651, AM234653, AM292919[9]), (FM180114-FM180181, FM178885-FM178951[24]); and the African Potamonautidae (AY042249[21]), (AY803554, AY803710, AY803690, AY919081, AY919086-AY919088, AY919126, AY919129, AY919132, AY919135[27]).

**Figure 3:**
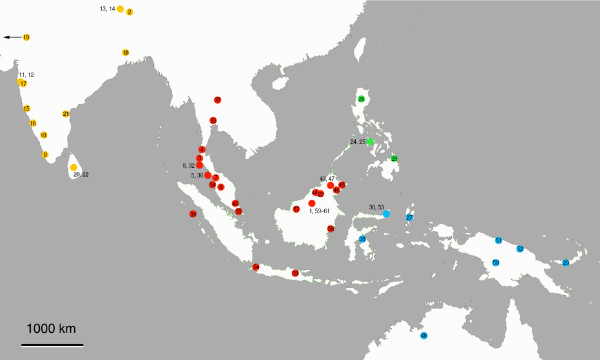
**Sampling of gecarcinucid species**. Map showing the sampling sites of the here included specimens, coloured according to the area encoding of the biogeographic analyses (yellow: India; red: Southeast Asia; green: Philippines; blue: Wallacea, New Guinea, Australia). Numbers correspond to the numbering of specimens in Fig. 2. For five specimens, originating from older collections or from the aquarium trade, only approximate locations are known, and thus are not shown here.

Our study includes 57 gecarcinucid species of 31 genera. These genera (55% of the gecarcinucid genus-level diversity) cover 60% of the currently recognized gecarcinucid species. As a sub-sample of the mitochondrial data given by Klaus et al. [24], the present study includes representatives of all gecarcinucid clades covering their whole range (Fig. 3). Only East Asian species are not included. However, these are closely related with the Southeast Asian genera *Sayamia *and *Siamthelphusa *that are included here [24].

As outgroups we included species of the other two Old World freshwater crab families, the Eurasian-North African Potamidae and the African-Madagascan Potamonautidae. Sequences were separated in two partitions, one for the H3 gene (318 bp) and one for the 16 S rDNA (558 bp), resulting in a total sequence length of 875 bp. Alignment was done with ClustalW [48] as implemented in the software BioEdit 7.0.9.0 [49] and adjusted manually.

A maximum likelihood analysis was conducted in the software RaxML 7.0.3 [50] with the alignment partitioned for the 16 S rDNA gene and all three codon positions of the H3 gene. A GTR+I+gamma model of sequence evolution was used for the final maximum likelihood search, as suggested by the AIC in jModelTest 0.1.1 [51], while for rapid bootstrapping (10^3 ^replicates) CAT approximation of rate heterogeneity is implemented in RaxML [52]. For Bayesian phylogenetic analysis and estimation of divergence times both partitions were unlinked and analysed in the software BEAST 1.5.2 [53]. After a preliminary series of test runs, the following models showed to fit best to the data: a relaxed, uncorrelated lognormal molecular clock model [54], a Yule tree prior, and a GTR+I+gamma model of sequence evolution. We ran the Markov chain for 10^8 ^generations, sampling 10^6 ^trees and discarding the first 10^5 ^as burn-in. Convergence of the Markov chain was investigated with Tracer 1.5 [55].

To estimate divergence times we conducted two Bayesian analyses under the above conditions, one with fossil calibration points and one with a discrete rate of evolution for the 16 S rDNA. We used three fossil calibration points: the oldest known potamid *Potamon quenstedti *found in South Germany, that we assume to be closer related to the Mediterranean *Potamon fluviatile *than to the Near East *Potamon persicum *(node *P. fluviatile *- *P. persicum*; c. 16.5 Ma [29]), a fossil of *Potamonautes *that was assigned to *P. niloticus *(node *P. niloticus *- *Platythelphusa armata*; 6 Ma [56]) and a claw fragment from the Siwalik beds in northern India related to the genus *Sartoriana *[29]. As the fossil site is situated within the range of extant *S. spinigera*, and far from *S. blanfordi *occurring in Pakistan (Balochistan) and Afghanistan, we assume a closer relationship with *S. spinigera *(node *S. spinigera *- *S. blanfordi*; Gecarcinucidae, 2.5 Ma [29]). To incorporate temporal uncertainty and expecting the MRCA at the respective nodes to predate the fossils, we assumed a gamma-distribution for the calibration points with the respective age as lower cut-off value and shape parameters of 4.0, 2.0 and 3.0 respectively. These different values for the shape parameter reflect different degrees of uncertainty of the exact age and taxonomic assignment of the fossils. Although the values have to be chosen arbitrarily, this approach is considered to be much more reliable than assuming a uniform or normal prior probability distribution for the nodes' ages [57]. The default value (1.0) for the scale factor was used.

We compared the results with the second analysis using a discretized relaxed molecular clock model (uncorrelated lognormal) with a rate of evolution for the 16 S rDNA partition of 0.88% per Ma (10% SD). This rate was calibrated for Jamaican sesarmids based on the closure of the Isthmus of Panama [58]. We calculated the Bayes factor between the calibrated and the discretized molecular clock model. The Bayes factor is the ratio of the marginal likelihoods between two hypotheses, whereby the marginal likelihoods are here estimated in Tracer. A common logarithm of the Bayes factor (log_10 _BF) larger than 0.48 indicates substantial, over 1.00 strong and above 1.48 very strong support for the respective hypothesis [59]. The comparison of the two hypotheses strongly supports the calibrated model of sequence evolution (log_10 _BF 1.69), thus giving an independent confirmation of the adequateness of the used fossil calibration points. The sesarmid rate of 16 S rDNA evolution (0.88% per Ma) nevertheless falls within the 95% HPD credibility interval of the 16 S rDNA rate of the calibrated model (0.64%-1.42% per Ma; mean value 1.02% per Ma). The 95% HPD of the rate of histone H3 is 0.12-0.26% per Ma with a mean of 0.19%.

### 0.4 Biogeographic analyses

To reconstruct ancestral ranges the maximum clade credibility tree inferred by the fossil calibrated model of Bayesian inference was used as phylogenetic input for DIVA and the DEC-model. The Bayesian approach was conducted with 10^3 ^post-burn-in trees to account for uncertainty in phylogenetic reconstruction. The gecarcinucid species were assigned to one of four discrete geographic areas: (a) the Indian Subcontinent, (b) Southeast Asia, including the Larger Sunda Islands that are situated on the continental shelf, (c) the Philippines, and (d) the gecarcinucid range east of the Wallace Line (Figs. 2 and 3). We did not take into account the distribution data of the outgroups, the potamoid families Potamidae and Potamonautidae. We have a fundamental reservation against inclusion of these to infer gecarcinucid biogeography. As long as the marine relatives of the Potamoidea remain unknown we can not exclude independent colonization of the limnic habitat by each potamoid family and consequent paraphyly of the Potamoidea. Thus we removed for the subsequent biogeographic analyses the outgroup taxa from the maximum clade credibility tree in Mesquite 2.72 [60].

Dispersal-vicariance analysis as implemented in the software DIVA [61] was conducted with maximally 10^3 ^alternative reconstructions kept at each node and the maximum number of ancestral areas left unconstrained. We are aware of the pitfalls and limitations of event-based biogeographic methods and consider this analysis as explorative [62,63]. We further analysed the data with a Bayesian approach to character evolution in BayesTraits multistate [64]. We let the analysis run for 5 × 10^7 ^generations, sampling 5 × 10^4 ^generations and discarding the first 5 × 10^3 ^samples as burn-in. To specify the range of values used to seed the prior distribution, we applied an exponential hyperprior with the mean from 0 to 80 and a rate deviation value of 0.1, resulting in a mean acceptance rate of 33.4% (SD = 8.6). We examined at each node if there is support for one area over another: we constrained the ancestral state of the node to one area and compared the harmonic means (an estimator of marginal likelihoods) between runs under different constraints by calculating the Bayes factor between them. As harmonic means can be unstable we repeated each run five times. To test for the direction of gecarcinucid dispersal we applied a parametric DEC approach [65,66] as implemented in the software Lagrange vers. 20091004 [67]. We calculated three models of gecarcinucid range evolution: an unconstrained hypothesis (H_0_), a model with only dispersal from India to Asia enabled (H_*I→A*_), and a third model allowing only dispersal from Asia to India (H_*A→I*_). We compared the resulting global maximum likelihoods at the root node and the AIC and Akaike weight between models (Table 2). For all three analyses the root age was set to the mean root age (47.23 Ma), adjacency of India with the Philippine range and the area East of the Wallace Line was restricted, and the rate parameters for dispersal and extinction were estimated by Lagrange.

### 0.5 Timing of the Indian-Southeast Asian faunal exchange

To approximate the timing of faunal exchange between India and Southeast, and to investigate if Indian and Southeast Asian species split before, during or after the final collision of India and Eurasia, we applied a model testing approach [59]. We set different time constraints for the basal nodes of the subfamilies Liotelphusinae and Parathelphusinae (where Indian and Southeast Asian species separate) and tested several hypotheses against each other: an Oligocene (25 Ma), Late Eocene (35 Ma) and Middle Eocene (45 Ma) age of the liotelphusine and parathelphusine MRCA. In addition, we calculated four models with the MRCAs of both subfamilies constrained to different ages, addressing the possibility that the two splits were not contemporaneous: before and during continental collision (45 and 35 Ma) and during and after this event (35 and 25 Ma). Not all theoretically possible combinations of time constraints could be calculated, as the initial tree likelihood in the Bayesian analyses dropped to zero (indicating that the respective prior model assumptions strongly contradict the data). We assumed a standard deviation of 20% to account for general temporal uncertainty. These time constraints (45, 35 and 25 Ma) fall within the 95% HPD credibility interval of the nodes' age estimates of the unconstrained analysis, and correspond to the time points for which paleogeographical reconstructions are given by Ali and Aitchison [17]. The constrained Bayesian analyses were conducted as described previously applying the calibrated relaxed molecular clock model. To evaluate the different hypotheses we calculated the Bayes factor between them in Tracer (Table 1).

## Authors' contributions

SK and MP designed the analyses, SK conducted the analyses and wrote the manuscript, all authors discussed the results and commented on the manuscript.
